# Nutritional Indicators of Bone Nonunion: A Systematic Review

**DOI:** 10.3390/jcm13216553

**Published:** 2024-10-31

**Authors:** Eleanor Christianson, Margaret Thomas, Sheila Sprague, Jessica Rivera, Andrew Chapple, Robert Zura

**Affiliations:** 1 Department of Orthopedic Surgery, Tulane University School of Medicine, New Orleans, LA 70112, USA; 2 Department of Orthopedic Surgery, Louisiana State University Health Sciences Center, New Orleans, LA 70112, USA; 3 Department of Surgery, McMaster University, Hamilton, ON L8S 4K1, Canada

**Keywords:** nonunion, nutrition, vitamin D, sarcopenia

## Abstract

**Background/Objectives**: Bone nonunion remains a clinical challenge in orthopedic surgery with significant impacts on mental and physical wellbeing for patients. There are several previously established risk factors of nonunion that are connected to nutrition, but this has yet to be substantially explored. This review seeks to assess all studies that present associations between nutrition and nonunion to understand the potential for clinical relevance in nonunion prevention. **Methods**: Case–control and cohort studies comparing nonunion risk based on nutritional factors were gathered through PubMed in July 2024. Data were extracted with dual verification through Covidence and assessed for bias using the Newcastle–Ottawa Scale. **Results**: A total of 21 studies were included in this literature review. Vitamin D deficiency was a significant risk factor of nonunion in six studies and not significant in six other studies. Albumin was significant in three of the five studies addressing this lab value. Iron deficiency anemia was significant in a study assessing its impact on nonunion. Calcium was not significant in the one study mentioned. ICD-10-coded malnutrition was significant in one of the two studies. Sarcopenia, nutritional care plans, and dietitian-diagnosed malnutrition were statistically significant clinical indicators for predicting nonunion, but food insecurity was insignificant. **Conclusions**: Vitamin D, calcium, albumin, iron deficiency anemia, sarcopenia, and clinically diagnosed malnutrition have all been associated with an increased risk of nonunion in observational studies and should be considered when preventing nonunion development.

## 1. Introduction

Bone nonunion remains a significant clinical challenge in orthopedic surgery. Large cohort studies have shown the nonunion rate to be approximately 5% and higher for certain populations and fracture types [1]. The US Food and Drug Administration (FDA) defines bone nonunion as any fracture that does not heal for nine months or shows no signs of healing for three months [2]. Nonunion creates heightened psychological stress and is associated with lower overall physical health in patients [3].

Although the exact etiology of nonunion is not established, the source is likely multifactorial. Many risk factors have been previously associated with nonunion including the type of fracture, treatment, underlying medical conditions (e.g., osteoporosis), and demographic and lifestyle factors (e.g., smoking, obesity.) Several of these risk factors are linked to nutrition, yet few studies have focused specifically on nutrition as a risk factor for nonunion. Both macro- and micronutrients including vitamins and minerals are critical for both bone growth, maintenance, and healing [4]. Deficiencies in these nutrients can have drastic impacts on bone health and have been shown to increase bone loss and fracture risk in various studies [4]. Since nutritional deficiencies are a demonstrated risk factor for other negative orthopedic outcomes, this creates the question of whether these deficiencies carry the same risk for nonunion development.

This review seeks to assess all studies that investigate associations between nutrition and nonunion with the overall goal of determining which tests and diagnoses may serve as useful clinical indicators in preventing nonunion after fracture.

## 2. Materials and Methods

### 2.1. Literature Search

This literature review was conducted by EC using PubMed searches for all relevant publications related to nutritional indicators of nonunion (Table 1). Initial query terms were chosen by EC based on nutritional indicators identified as relevant for bone health and fracture healing [4]. Additional searches with topics connected to nutrition were included by EC to ensure the complete inclusion of all relevant studies.

### 2.2. Eligibility Criteria

Studies were excluded if they were biochemical studies, literature reviews, animal models, and case studies along with abstracts and studies with a sample size of under 20 people. For full text review exclusion criteria, studies were excluded if nonunion was not a main outcome, if the outcome compared different types of nonunion, if there was an underlying endocrine disorder for all participants including controls, or if nutritional indicators were not a main exposure of the study. Case–control and cohort studies with sample sizes of 20 or more people comparing nonunion risk based on nutritional factors were included in this study.

### 2.3. Article Screening

All studies identified from the PubMed searches were initially included by EC. Covidence identified and removed duplicates. All subsequent screening phases were performed by MT and EC independently to avoid bias and prevent exclusion of relevant studies. In the abstract screening and full text review phases, studies that did not meet the eligibility criteria were excluded.

### 2.4. Data Extraction and Analysis

Data extraction was conducted through Covidence independently by MT and EC [5]. The significance, risk ratios, fracture type, population, and data of various nutritional values were extracted from full text by EC and MT and compared for accuracy. These values were used to assess the impact of each nutritional factor on nonunion. Any conclusions discussed in this text were based only on the information clearly presented in the included texts. Based on an in-depth review of literature, articles were grouped in the analysis based on vitamin D, other serum values, and nutrition-related diagnoses. Texts addressing both or all of these sections were included in their relevant discussions and not limited to one section.

### 2.5. Bias Assessment

All studies included were quality assessed for potential biases using the Newcastle–Ottawa Scale which is for cohort and case–control studies. Each study was reviewed independently by EC and MT.

## 3. Results

**Figure 1:**
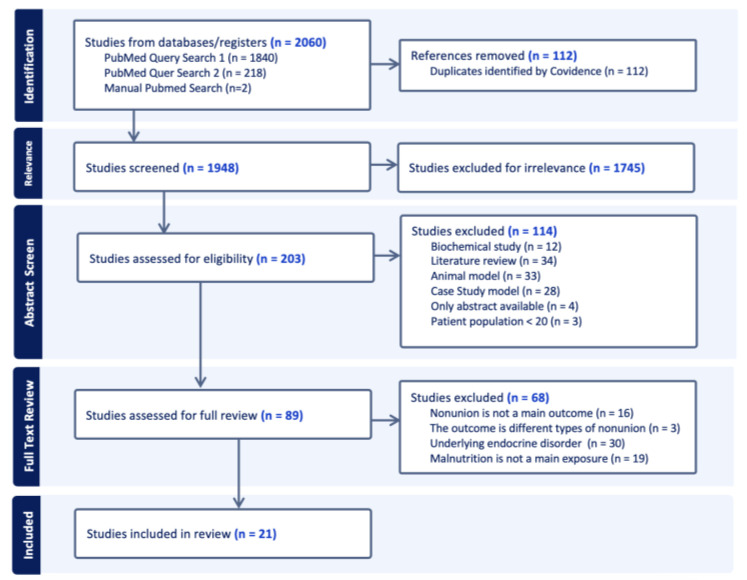
PRISMA chart which summarizes the literature review process.

The three PubMed searches resulted in the initial inclusion of 2060 studies (Figure 1). Covidence removed 112 studies that were duplicates. Additionally, 1745 irrelevant studies were excluded before abstract screening. Of the 203 remaining articles, there were 12 biochemical studies, 34 literature reviews, 33 animal models, and 28 case studies. There were four studies with only abstracts available, and three studies where the sample size was below 20. This resulted in 89 studies for full text review. In full text review, sixteen studies did not have nonunion as a main outcome; three studies had different types of nonunion; thirty studies included cohorts with underlying metabolic disorders; and, lastly, nineteen studies did not have nutritional indicators as a main outcome.

Therefore, 21 case–control and cohort studies were included in this review. These studies were subdivided into three main themes including vitamin D (twelve studies), other serum lab values (seven studies), and malnutrition-related diagnoses (six studies). Some studies addressed two or all of these themes. All included studies had a relatively low risk of bias based on the Newcastle–Ottawa Scale assessment.

### 3.1. Vitamin D Studies

There were 12 studies identified that focused on vitamin D (Table 2). Of these, six papers found significant results associating vitamin D deficiency to nonunion. Zura et al. [1] performed a study analyzing the risk factors for nonunion across 18 different bones in adults and found vitamin D deficiency to be a significant risk factor, showing a univariate OR of 1.44 (CI: 1.34, 1.54) and a multivariate OR of 1.14 (1.05–1.22; *p* < 0.001). In a similar study, Zura et al. [6] performed a separate inception cohort study of 237,033 pediatric patients that analyzed vitamin D among various others as a risk factor for nonunion. The results showed a significant univariate OR of 3.98 (CI: 2.70, 5.84) and a multivariate OR of 2.91 (1.91, 4.42; *p* = < 0.0001). These paired studies suggest that the associated risk factors for nonunion are similar for pediatric and adult populations, but the correlation of vitamin D deficiency and nonunion seems to be stronger in pediatric populations.

Ravinda et al. [7] measured preoperative vitamin D levels of 133 adults undergoing elective spinal fusion and found a significant difference of vitamin D levels with nonunion rates of 20% and 38% in adequate and deficient vitamin D levels, respectively (*p* = 0.063). There was also an increased OR of 3.45 for nonunion (*p* = 0.045) and longer times to fusion (*p* = 0.001) [7]. Zhou et al. [8] assessed vitamin D levels for cervical fusion rates of spondylotic spines and found that serum vitamin D was a significant predictor of nonunion. Ramanathan et al. [9] assessed patients who underwent ankle fusion procedures and evaluated the incidence of reoperation with available vitamin D levels. A significant association was found with zero of thirty patients in the control cohort and four of seventeen of the vitamin D deficient cohort and underwent reoperation secondary to nonunion (*p* = 0.013) [9]. In a case–control study of 58 patients conducted by Moore et al. [10], it was found that patients with vitamin D deficiency were 8.1 times more likely to have a nonunion (*p* = 0.02, CI: 1.996–32.787).

Six studies did not find a significant association between vitamin D levels and fracture healing. Using a cohort of musculoskeletal trauma patients, one study evaluating the medical complications and preoperative vitamin D levels of 373 patients found an insignificant association (0.72 (CI: 0.41, 1.26; *p* = 0.25)) [11]. Donnaly et al. [12] found no association between vitamin D levels and rates of postoperative pseudarthrosis, revision, or hardware complications after lumbar spinal fusion across a 150-patient population (*p* > 0.05). In Clark et al. [13], thirty-three patients undergoing corrective distal radial malunion were investigated; seven patients had a nonunion, and vitamin D deficiency was not found to be a significant risk factor of nonunion.

Two studies investigated long bone nonunion of the lower extremities. The first included 370 patients with tibia and fibula fractures that required surgical intervention, and 90% (n = 210) had vitamin D insufficiency; however, there was no statistical difference in union rates [14]. The second study investigated patients with femoral neck fractures that required cannulated screws. Of these patients, 29% required reoperation and vitamin D status was not a significant predictor of nonunion [15]. One study investigated the effect of implementing a one-time 100,000 IU oral vitamin D supplement to patients with vitamin D deficiency (<20 ng/mL) and vitamin D insufficiency (<30 ng/mL), but these treatments were found to have no significant effect on nonunion between the treatment and control group [16]. Overall, low vitamin D levels were found to be associated with nonunion in certain populations, but not others.

### 3.2. Other Serum Values

Albumin is a well-established marker of malnutrition that was analyzed in five of the studies in this literature review (Table 3). Bajada et al. [17] retrospectively assessed the preoperative serum albumin of 111 undisplaced intracapsular hip fractures treated with cannulated screws and found that patients with fixation failure had on average 5 g/mL lower albumin levels compared to properly healed cases (*p* = 0.02); the odds ratio of albumin in a multivariate analysis was 0.188 (CI: 0.046–0.765, *p* = 0.008). The proposed mechanism for this relationship was based on the connection between low bone mineral density and hypoalbuminemia along with increased risk of falls in malnourished people [17]. In a retrospective review of 251 femoral neck fractures, Riaz et al. [18] showed that lower preoperative albumin levels were a significant independent risk factor associated with fixation failure including nonunion and explained that it could be a helpful tool for deciding between fixation or arthroplasty (*p* = 0.01). They cited similar reasoning for this connection as Bajada’s explanation. In the Hendrickson et al. study [11], 373 patients with operative fixation were screened by a dietitian, and albumin labs were drawn if they were at medium to high risk; 41% of patients had hypoalbuminemia (<3.5 mg/dL) and the OR for complication risk including nonunion was 1.79 (*p* = 0.045). Hendrickson proposed that albumin may be increased in these cases more so due to the movement of plasma during the acute phase reaction of trauma versus any connection with nutrition, citing that current nutritional guidelines do not recommend albumin as a reliable clinical measure of nutrition. Lastly, two other studies did not find albumin to be a significant risk factor, including Chen et al. [15], who reviewed 204 nonunion cases, and Gregerson et al. [19], who assessed nonunion risk factors for 322 operative femoral neck fractures (*p* > 0.05).

Iron deficiency anemia is a nutritional deficiency that was shown to be a significant predictor of nonunion in two of the studies included in this literature review (Table 3). Anderson et al. published that in a cohort of 9482 metatarsal fractures, fractures with delayed healing were associated with higher rates of iron deficiency anemia (*p* = 0.016). In Sanchez et al. [20], 1020 nonunion cases of proximal humerus fractures were compared to controls, and iron deficiency anemia had an OR of 1.32 6 months after open reduction and internal fixation (ORIF) (*p* < 0.0001). Sanchez et al. proposed that iron deficiency anemia has a role in nonunion due to osteoporosis development through a reduction in vitamin D activation and hypoxia.

Lastly, Liu et al. [21] analyzed different lab values in 170 posttraumatic osteomyelitis and 130 aseptic nonunion patients in comparison to 168 controls (Table 3). There was no statistical difference between the nonunion and control groups for serum calcium (*p* = 0.197). Serum phosphorus levels of control (1.24 mmol/L) were lower compared to nonunion cases (1.29 mmol/L) (*p* = 0.011).

### 3.3. Malnutrition and Related Diagnoses

In this literature review, six papers assessed the connection between various nutritional diagnoses with the risk of nonunion (Table 4). Zura et al. [1] retrospectively analyzed 209,330 fractures and did not find a significant association between malnutrition, measured by record of an International Classification of Diseases (ICD)-coded diagnosis, and nonunion (*p* > 0.05). Wilkinson et al. [22] assessed 178,283 femur fractures by dividing them based on the presence of ICD-9 and 10-coded malnutrition; in this case, malnutrition significantly increased the risk of nonunion (1.89; 95% CI: 1.69–2.11; *p* < 0.0001). Vander Voort et al. [23] measured sarcopenia, a diagnosis related to malnutrition, based on the psoas index (women <3.85 m^2^/m^2^, men <5.45 cm^2^/m^2^) in 111 patients with operative tibia or ankle fractures; the risk ratio for sarcopenia and nonunion was 2.42 (CI: 1.08–5.43, *p* = 0.0314).

Vo et al. [24] took a public health approach and compared patients with fractures who were food insecure to others who were not. They did not find a statistically significant association to nonunion. As mentioned briefly before, Hendrickson et al. assessed the nutritional status of 373 fracture patients via a malnutrition questionnaire, lab values, and a clinical consultation with a dietitian. While the questionnaire score did not yield a significant result, the dietitian-diagnosed malnutrition was a significant predictor of complications including nonunion (OR 3.49, *p* = 0.017) [11]. Hendrickson et al. noted that clinical diagnosis for malnutrition was less common than abnormal serological values, and it also had a sensitivity of 21% and a specificity of 87%. Finally, Lu et al. compared the outcomes of 40 patients with conventional care to 40 patients with a multidisciplinary care team that included a nutritionist who provided supplemental nutrition to malnourished patients. Patients with additional care had better rates of bone healing and increased body weight and albumin levels (*p* < 0.05) [25]. The authors explained that the addition of a nutritionist aided in wound healing.

**Figure 2:**
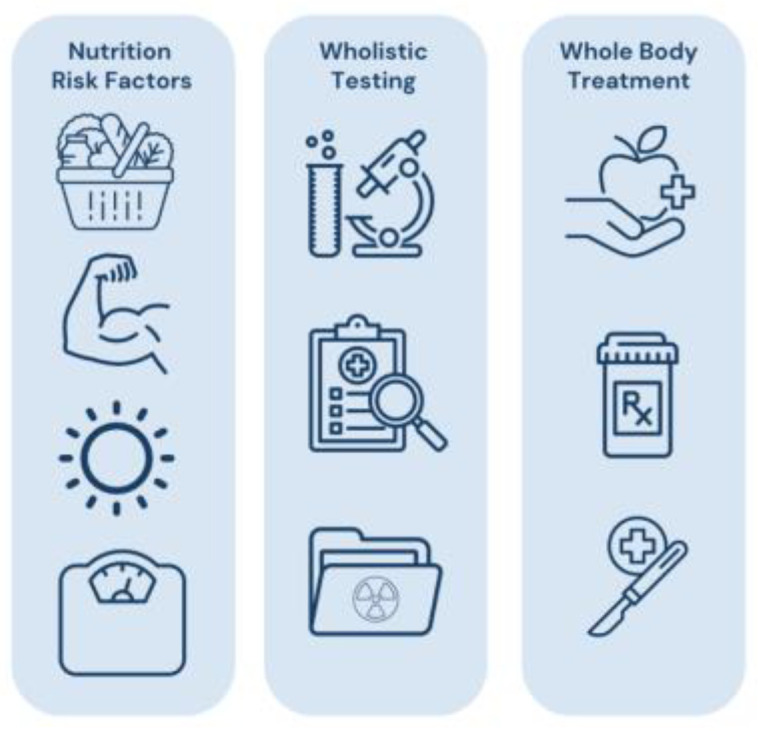
An inclusion of nutrition in orthopedic practice. Several nutritional risk factors may impact initial fracture and risk of nonunion. Labs, questionnaires, and imaging can be used together to create a clinical picture. From there, surgical intervention and supportive nutritional care can prevent nonunion.

Several of these studies show associations between nutritional values and diagnoses with nonunion. While there is some uncertainty, it is an area that can be further incorporated into the orthopedic setting through the integration of testing for factors related to nutrition when assessing patients. Nutritional supplementation can also potentially support further fracture healing to prevent nonunion. Figure 2 shows a depiction of nutritional integration into an orthopedic care plan. 

## 4. Discussion

### 4.1. Summary

The purpose of this literature review was to assess the association between nonunion development and nutritional factors in various fracture patient populations. While nutrition has been assessed in several studies on fracture risk, few studies mentioned nutrition in relation to nonunion risk. Vitamin D, serum lab values including albumin, and iron deficiency anemia, and nutritional diagnoses such as sarcopenia or malnutrition are the main topics in previous publications in relation to nonunion specifically. The studies in this literature review have varying levels of significance for the association between nonunion and nutritional factors, requiring further research on this topic.

### 4.2. Vitamin D

The relationship between nonunion and vitamin D levels was most frequently studied in this literature review. There was an inconsistency in the significance of this relationship, which can likely be attributed to issues of statistical power, a potentially weak but significant relationship between the two factors, and less severe deficiencies present within the selected patient populations. For bone mineralization and subsequent union to occur after a fracture, there must be sufficient available calcium and phosphate to be secreted into the osteoid layer of the healing bone. Without adequate vitamin D, the body can absorb only 10–15% of calcium, rather than the 30–40% absorption achieved with sufficient vitamin levels [26]. This deficiency is highly prevalent; an analysis of the Third National Health and Nutrition Examination Survey by Khazai found that 61% of white and 91% of Black Americans were considered vitamin D deficient with a serum 25[OH]D concentration of <32 ng/mL [26].

While *The Journal of Endocrinology* cites insufficiency as 21–29 ng/mL and deficiency as <20 ng/mL, there is variation in the terminology used in these studies [27]. Zhou describes normal vitamin D levels as within the range of 30–100 ng/mL. The study by Donnally et al., which found no significant results, defined vitamin D deficiency as lab levels below 31 ng/mL. Another study with no significant results, conducted by Clark et al. [13], states that patients are vitamin D deficient but does not define this term. The lack of significance is likely due to the small margins of difference between the control and deficient groups. This idea is reinforced by the Zura et al.’s 2016 paper [1], which evaluated 309,330 adult fractures. Vitamin D deficiency was a significant risk factor, but the univariate odds ratio (OR) was weaker with values of only 1.44 (CI: 1.34, 1.54). The other Zura et al. (2018) study [6] shows a higher OR of 3.98 (CI: 2.70, 5.84), but this study involved a pediatric population, and the higher demand for calcium due to the developing skeleton must be considered. Of the six papers that reported no significant effect, the sample sizes ranged from 33 to 468. Given the likely multifactorial nature of nonunion etiology, vitamin D may be more impactful in some cases of nonunion and a less impactful in others. Age, fracture type, muscle mass, and other nutritional deficiencies may play a larger role in nonunion development. Overall, the fluctuation in results is likely attributable to a combination of small sample sizes, mildly deficient patient populations, and a weakly positive correlation.

### 4.3. Other Serum Values

Only one study investigated serum calcium levels related to nonunion, and there was no significant correlation between nonunion and serum calcium levels, but this is not an adequate picture of the role of calcium. Part of the exclusion criteria for this review was studies that investigated the role of exogenous parathyroid hormones since they are not considered a part of dietary intake or multivitamins. For consistency, all other studies investigating the role of parathyroid hormones and calcium homeostasis were excluded. This resulted in a limited view of the role of calcium in fracture nonunion in this review.

Of the twenty-one studies included in this literature review, five provided insights into the relationship between serum albumin levels and nonunion. Albumin has historically been used as a biomarker for nutritional assessment due to its sensitivity to changes in protein status and liver function. While serum albumin concentrations are indicative of protein synthesis capacity, there have been criticisms for the accuracy of this metric due to its lack of specificity and long half-life [28]. Hendrickson et al. provided additional insight on the utility of this test more as a marker of trauma response versus malnutrition, which is more of a chronic process. In this review, three of the five studies analyzing albumin found a significant difference in nonunion rates for patients with hypoalbuminemia. It is likely that while imperfect, albumin may be able to provide insight into a patient’s overall health, especially when included as a value in a nutrition score like the prognostic nutrition index (PNI). This score was recently shown as a mortality predictor in hip fracture patients and could potentially be applied to nonunion risk [19]. While albumin alone has mixed success in predicting nonunion, it may be a helpful indicator when measured within a panel of nutritional tests.

Only one study in this review analyzed the relationship between iron deficiency anemia and nonunion and found a statistically significant association between the two. Anderson et al. published that in a cohort of 9482 metatarsal fractures, fractures with delayed healing had higher rates of iron deficiency anemia (*p* = 0.016). Iron deficiency anemia has also been linked to increased stress fracture, implant failure, and surgical complications in various cohorts [29,30,31]. This could be explained by the fact that iron deficiency anemia is connected with the loss of bone mineral density, which increases the risk of bone fragility, but causality has not been definitively established [32]. Iron is a key factor for mitochondrial metabolism, which plays a part in both osteoblastic and osteoclastic differentiation in the body; iron is also a critical factor in preventing hypoxic conditions, producing collagen, and regulating vitamin D activation, which are all important processes for bone healing [32]. For these reasons, it is logical that iron deficiency anemia is an important indicator for nonunion risk and should be screened for in fracture cases.

### 4.4. Nutrition-Related Diagnoses

Six of the twenty-one studies included in this review explored associations between nutrition and nonunion through various metrics including ICD-10 codes, public health surveys, and nutritionist assessments. ICD-10-coded malnutrition was significant in Wilkinson et al. but not in Zura et al., 2016 [1]. This is likely due to the differences in the age inclusion of each study population; Zura et al. only included patients between 18 and 63 years of age, while Wilkinson included a patient cohort of 65 years and older. Malnutrition may play a greater role in nonunion for older patients since this population is more likely to be malnourished and has a higher risk for fracture as well [33]. Malnutrition is often the underlying cause of sarcopenia, which makes the results of Vander Voort et al. particularly relevant for this review [34]. The relationship between frailty, nutrition, and sarcopenia has been well described previously [35]. The logical extension to nonunion is that a continued state of nutritional deficit and frailty prevents the patient’s bone from properly healing. This is a helpful indicator that should be screened for, especially in elderly populations. Vo et al. [24] did not find statistical significance when analyzing food insecurity, but this metric may not have the best specificity for metabolic malnutrition. The food insecurity screening questionnaire asks about access to food in the past 12 months, which might not be descriptive of the patient’s current nutritional state at the time of fracture admission. Both Hendrickson et al. and Lu et al. underscore the importance of incorporating a multidisciplinary care approach for orthopedic patients who are nutritionally compromised. In Hendrickson et al [11], dietitian-diagnosed malnutrition was the best predictor of complication risk compared to questionnaires and lab values. In Lu et al. [25], patients with nutritional support had better outcomes as well. Nutritionist consultation and support allows for the proper identification of at-risk patients and adequate supplementation to aid in proper healing progression.

Current nutritional practices for orthopedic surgeons mainly include vitamin D supplementation, but this is wholly inadequate in addressing the nutritional deficits that nonunion cases often present with. Nutritional lab values, screening questionnaires, and nutritional consultation are all useful clinical tools that can decrease risk for fracture patients. Nonunion risk may be impacted by a variety of factors beyond nutrition; the multifactorial nature of this process makes prevention even more complex. Poor nutrition is likely one of several risk factors that cause nonunion. Importantly, nutrition may be the one of the more modifiable risk factors that can be improved with minimal effort and expense. By addressing nutritional deficits at the time of fracture, surgeons can mitigate some risk later in the healing process which improves patient quality of life, avoids cost, and lowers risk of nonunion.

### 4.5. Strengths, Limitations, and Future Directions

This literature review had a broad initial inclusion of articles to make sure all relevant articles were included. After this inclusion, rigorous exclusion criteria were adopted to identify only the relevant articles. Dual screening prevented bias and improper exclusion or inclusion of articles. This literature review was limited by the availability of research on nutrition with nonunion specifically. After the initial literature searches and inclusion into Covidence, additional articles were not screened for, leading to the potential exclusion of more recently published articles. Lastly, few articles in this literature review had similar methods and statistical impact, which makes comparison between papers difficult.

This review prompts the need for further research on (a) the extent of impact on nonunion development in comparison to other nonunion risk factors, (b) the variability of impact on different types of nonunion or bone involvement, (c) the potential difference in nutrition risk factors for nonunion based on age and gender, and (d) the determination of lab values that are most important in identifying nutritional risk for nonunion.

## 5. Conclusions

This review assessed the association between nutritional indicators and nonunion. Vitamin D, calcium, albumin, iron deficiency anemia, sarcopenia, and diagnosed malnutrition have been associated with an increased risk of nonunion in observational studies; however, the literature is highly variable and inconsistent. Given the multifactorial nature of nonunion, it is likely that poor nutrition is one of several variables that can lead to nonunion development. Further research on the utility of nutritional screening and supplementation is needed to create nutritional recommendations that aid orthopedic surgeons in counselling their patients on nutrition and reducing the risk of nonunion. 

## Figures and Tables

**Table 1:** PubMed search information. All PubMed inquiries used for this review. This includes the 112 duplicates that were identified by Covidence and automatically removed before abstract screening.

Search	Query Terms	Studies Added	Date Searched
PubMed Search 1	(nonunion) AND ((calcium) OR (vitamin D) OR (25-dihydroxycholecalciferol) OR (calcitriol) OR (sodium) OR (phosphorus) OR (copper) OR (zinc) OR (manganese) OR (magnesium) OR (vitamin K) OR (vitamin B6) OR (DHA) OR (EPA) OR (iron) OR (potassium) OR (magnesium) OR (folic acid) OR (vitamin C) OR (vitamin A) OR (vitamin B12) OR (lysine) OR (proline) OR (bisphosphonates) OR (protein) OR (carbohydrates) OR (lipids)) data	1840	2 July 2024
PubMed Search 2	2 papers were manually imported based on previous knowledge of literature that were not included in the previous search.	2	2 July 2024
PubMed Search 3	(nonunion) AND ((nutrition) OR (malnutrition) OR (nutritional deficiency) OR (sarcopenia) OR (Vitamin) OR (nutrients))	218	3 July 2024

**Table 2:** Vitamin D studies. All studies that included vitamin D (vit D) as a main exposure or risk factor of nonunion.

Paper	Study Design	Bone Studied	Result	Significance
Zura, 2016 [1]	Risk factors analyzed for nonunion for 309,330 fractures in 18 bones.	Metatarsal, radius, ankle, metacarpals, trunk, tarsal, humerus, tibia, ulna, clavicle, scaphoid, patella, pelvis, fibula, femur	Nonunion rate was 4.9%. Nonunion risk increased with vitamin D deficiency (OR, 1.14; *p* < 0.001).	significant
Zura, 2018 [6]	Risk factors analyzed for nonunion in 237,033 pediatric fracture patients.	Metacarpal, radius, ankle, patella, ulna, fibula, pelvis, clavicle, humerus, femur, tibia, metatarsal, tarsal, scaphoid	Nonunion rate: 0.85%. Multivariate analysis shows deficiency has significant OR (*p* < 0.001).	significant
Ravindra, 2015 [7]	Vit D levels of 133 adults pre-elective spinal fusion (<20 ng/mL = deficient) to determine predictor of nonunion.	Cervical, thoracic, and lumbar vertebrae	Nonunion rate: 20% adequate Vit D level vs. 38% deficient patients (*p* = 0.063). Time to fusion longer in the deficient group (12 vs. 6 mo., *p* = 0.001). Multivariate analysis vit D deficiency is significant (OR 3.45, *p* = 0.045).	significant
Zhou, 2022 [8]	Vit D levels of 58 nonunion and 692 union cervical spondylotic cases measured	Cervical vertebrae	Serum Vit D (OR = 0.81, *p* < 0.001) was a significant predictor of nonunion.	significant
Ramanathan, 2022 [9]	Preop Vit D levels of 47 patients were split into deficient (<30 ng/mL) vs. normal (31–80 ng/mL) and reoperation for nonunion was assessed.	Ankle	The deficient group (n = 17; 36.2%) vs. normal (n = 30; 63.8%) Vit D levels were 16.9 and 46.4 ng/mL. Reoperations for nonunion occurred only in the deficient cohort (23.5%; *p* = 0.013).	significant
Moore, 2017 [10]	29 nonunion and 29 union patients with elective foot/ankle reconstruction were matched to assess nonunion risk factors including vit D.	Foot and ankle	Patients with vit D deficiency/insufficiency were 8.1 times more likely to have nonunion (*p* = 0.02, CI: 1.996–32.787).	significant
Hendrickson, 2019 [11]	373 patients with operative fracture screened using questionnaire and dietitian. If moderate-to-high risk, 25(OH) Vitamin D measured preoperatively.	Clavicle, scapula, shoulder, humerus, radius, forearm, pelvis, acetabulum, knee, patella, tibia, foot, ankle	Of 373 patients, 17% were at risk. Vit D was not a significant predictor of nonunion.	not significant
Donnally, 2019 [12]	Pre and postop vit D levels measured in 150 patients for impact on rates of postop pseudarthrosis, revision, or complications.	Lumbar vertebrae	Vit D levels were not significantly associated with rates of postoperative pseudarthrosis, revision, or hardware complications (*p* > 0.05).	not significant
Clark, 2021 [13]	Vit D levels of 33 patients with corrective osteotomy for distal radius malunion measured to predict nonunion.	Radius	Seven patients (21%) had nonunion after osteotomy. Vit D deficiency is not significant.	not significant
Gudeman, 2023 [14]	Vit D deficiency (<30 ng/mL) of 370 patients with operative tibia and fibula fractures to predict nonunion.	Tibia, fibula	98% (n = 210) of fractures had vit D insufficiency with a median of 22.7 ng/mL. No statistical difference between union rates.	not significant
Gregersen, 2015 [15]	Vit D of 322 femoral neck fractures fixed with cannulated screws were assessed.	Femur	29% underwent reoperation. Vit D was not a significant nonunion predictor.	not significant
Haines, 2017 [16]	100 vitamin D deficient/sufficient (<20 and <30 ng/mL) patients with long bone fractures; 50 received single dose of vitamin D (3) orally (100,000 IU) within two weeks of injury, 50 were placebo.	Humerus, femur, tibia	The initial median vit D levels were 16 ng/mL in both groups (*p* = 0.885). Nonunion rate was 4% (two per group).	not significant

**Table 3:** Other serum lab value studies. All studies that assessed serum values other than vitamin D.

Paper	Study Design	Bone Studied	Result	Significance
Bajada, 2015 [17]	Albumin levels measured for 111 patients with undisplaced intracapsular hip fractures treated with cannulated screws.	Femur	16% of fixations failed and had a significantly lower albumin (35 g/L vs. 40 g/L, *p* = 0.02) than non-failure patients becoming independent risk factors.	significant
Riaz, 2016 [18]	Preoperative albumin levels of 251 undisplaced intracapsular femoral neck fracture patients measured to assess fixation failure including nonunion.	Femur	12 (5%) patients had fixation failure from nonunion. Low serum albumin levels were significantly associated with failure (*p* = 0.01).	significant
Hendrickson, 2019 [11]	373 patients with operative fracture screened by dietitian. If moderate-to-high risk, albumin, transferrin, and total lymphocyte count measured.	Clavicle, scapula, shoulder, humerus, radius, forearm, pelvis, acetabulum, knee, patella, tibia, foot, ankle	Of 373 patients, 17% were at risk, ~50% of patients had 1+ serum deficiency. Complications occurred in 19% of patients. Hypoalbuminemia (OR 1.79, *p* = 0.045) was significant.	significant
Gregersen, 2015 [15]	Vit D and albumin of 322 operative femoral neck fractures assessed for outcomes including nonunion.	Femur	After 2 years, 29% underwent reoperation. Albumin was not significant.	not significant
Chen, 2024 [19]	Nonunion risk factors of 204 patients with defective bony nonunion compared to controls.	Tibia, humerus, fibula, femur, radius, ulna	Serum calcium levels were lower in the nonunion group (*p* = 0.01). Albumin was not significant. Glucose levels were higher in the nonunion group (*p* = 0.01).	not significant
Sanchez, 2023 [20]	ORIF for PHFs were divided into nonunion (n = 1020) and union (n = 51,209) to compare risk factors.	Humerus	Iron deficiency anemia (OR: 1.32; *p* = 0.0001) was significant for nonunion within 6 months of ORIF for PHF.	significant
Liu, 2021 [21]	Preoperative calcium, phosphorus, and PLT were collected for 468 patients (170 posttraumatic OM, 130 aseptic bone nonunion, 168 controls).	Not specified	No statistical difference between ABN and control for serum calcium (*p* = 0.197). Serum phosphorus level of control (1.24 mmol/L) were lower compared to ABN (1.29 mmol/L) (*p* = 0.011)	not significant

**Table 4:** Nutritional diagnoses studies. All studies that assessed nutrition-related diagnoses.

Paper	Study Design	Bone Studied	Result	Significance
Zura, 2016 [1]	Risk factors analyzed for nonunion for 309,330 fractures in 18 bones.	Metatarsal, radius, ankle, metacarpals, trunk, tarsal, humerus, tibia, ulna, clavicle, scaphoid, patella, pelvis, fibula, femur	Malnutrition was not significant.	not significant
Wilkinson, 2022 [22]	178,283 patients (65+) with operative femur fractures divided into malnutrition compared to non-malnourished cases.	Clavicle, scapula, humerus, radius, forearm, pelvic ring, femur, patella, tibia, foot, ankle	Patients with malnutrition are at increased risk of nonunion (1.89; 95% CI 1.6946–2.1095; *p* < 0.0001).	significant
Vander Voort, 2020 [23]	111 patients with operative fixation of open tibia/ankle fractures. Sarcopenia = psoas index at the L3 pedicle with <3.85 (women) and <5.45 cm^2^/m^2^ (men).	Tibia, ankle	16/100 (16%) patients had sarcopenia. Nonunion occurred in 6 patients with sarcopenia (38%) and 12 without (18%) (RR = 2.42, CI = 1.08–5.43, *p* = 0.0314).	significant
Vo, 2024 [24]	Food insecurity screening surveys of 100 (18+) patients surgically treated for fracture divided into food insecure and non-food insecure.	Not specified	37% were food insecure. There were no differences for nonunion risk.	not significant
Hendrickson, 2019 [11]	Patients (18+) with operative fracture screened using a malnutrition screening questionnaire (MSQ) and assessed by dietitian if moderate-to-high risk. Lab values measured preoperatively.	Clavicle, scapula, shoulder, humerus, radius, forearm, pelvis, acetabulum, knee, patella, tibia, foot, ankle	17% had increased risk, 4.3% were clinically malnourished from dietitian. 19% had complications including nonunion. Dietitian-diagnosed malnutrition was predictor (OR 3.49, *p* = 0.017). MSQ score was not correlated.	significant
Lu, 2024 [25]	80 patients with infectious bone defects had multidisciplinary team with nutritionist that provided oral, NG tube, or IV nutrition to malnourished patients. They were compared with 40 people without traditional care.	Tibia, femur	Delayed bone healing was higher in conventional care group (*p* = 0.006) with 27.5% compared to 5%. Changes in body weight, albumin, pre-albumin, hemoglobin, and sodium were higher in the MDT group (*p* < 0.05).	significant

## Data Availability

All data can be accessed through PubMed searches. Any information is available upon request.
